# Review Article: Liver Transplantation for Hepatocellular Carcinoma in the Era of Immune Checkpoint Inhibitors

**DOI:** 10.1111/apt.70333

**Published:** 2025-08-13

**Authors:** Anand V. Kulkarni, Amit G. Singal, K. Rajender Reddy

**Affiliations:** ^1^ Department of Hepatology AIG Hospitals Hyderabad India; ^2^ UT Southwestern Medical Center Dallas Texas USA; ^3^ Division of Gastroenterology and Hepatology University of Pennsylvania Philadelphia USA

**Keywords:** delisting, immunotherapy, liver cancer, living donor liver transplantation

## Abstract

**Background:**

Ablation, surgical resection and liver transplantation (LT) are curative therapies for patients with hepatocellular carcinoma (HCC). Milan Criteria and University of California San Francisco Criteria are traditionally accepted for liver transplantation, with the expectation of favourable outcomes. In recent years, immune checkpoint inhibitors (ICI) have revolutionised the management of unresectable HCC (uHCC) and are now considered first‐line systemic therapy.

**Aims:**

In this narrative review, we aimed to comprehensively discuss the role of ICIs in the peri‐transplant period, with the goal of enhancing the chances of a successful LT for advanced HCC while also decreasing the risk of recurrence post‐LT.

**Methods:**

A search of PubMed and manual screening of references was performed to identify studies evaluating ICIs in the context of LT, and relevant articles were included.

**Results:**

ICIs can achieve complete response and ultimately provide long‐term survival in a subset of patients. There has been an exponential increase in the use of these drugs, and increasing interest in the use of combination locoregional therapies plus ICIs as a strategy for downstaging or bridging to LT. While there can be objective responses with ICI therapy, there are potentially serious adverse events, including immune‐mediated liver injury and enhanced risk of infections in the pre‐LT period. Rejections and recurrence post‐LT are relevant in the context of ICIs, while endeavouring to downstage or bridge HCC pre‐transplant.

**Conclusions:**

ICI therapy is nuanced during the peri‐transplant period and should therefore be selectively used in specific patients rather than being used ubiquitously.

## Graphical abstract

ICI is now the first‐line therapy for unresectable HCC and has revolutionised the management of advanced HCC and is now emerging as a modality for downstaging or bridging. The ideal timing of transplant after ICI should be at least 52 days.

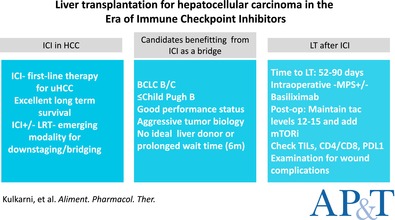

**Abbreviations**
AFP: alpha‐fetoprotein
BCLC: Barcelona Clinic Liver Cancer
DCP: des‐ gamma carboxyprothrombin
HBV: hepatitis B virus
HCV: hepatitis C virus
iRAE: immune‐related adverse events
LT: liver transplantation
LRT: locoregional therapy
LDLT: living donor liver transplantation
MC: Milan criteria
PIVKA: protein‐induced vitamin k absence
PET: positron emission tomography
PVT: portal vein thrombosis
RFS: recurrence‐free survival
SBRT: stereotactic body radiation therapy
STRIDE: single dose of tremelimumab plus repeated doses of durvalumab
TACE: transarterial chemoembolisation
TARE: transarterial radioembolisation
TKIs: tyrosine kinase inhibitors
uHCC: unresectable hepatocellular carcinoma
UCSF: University of California San Francisco
UNOS‐DS: United Nations organ sharing downstaging criteria

## 1 Introduction

**FIGURE 1:**
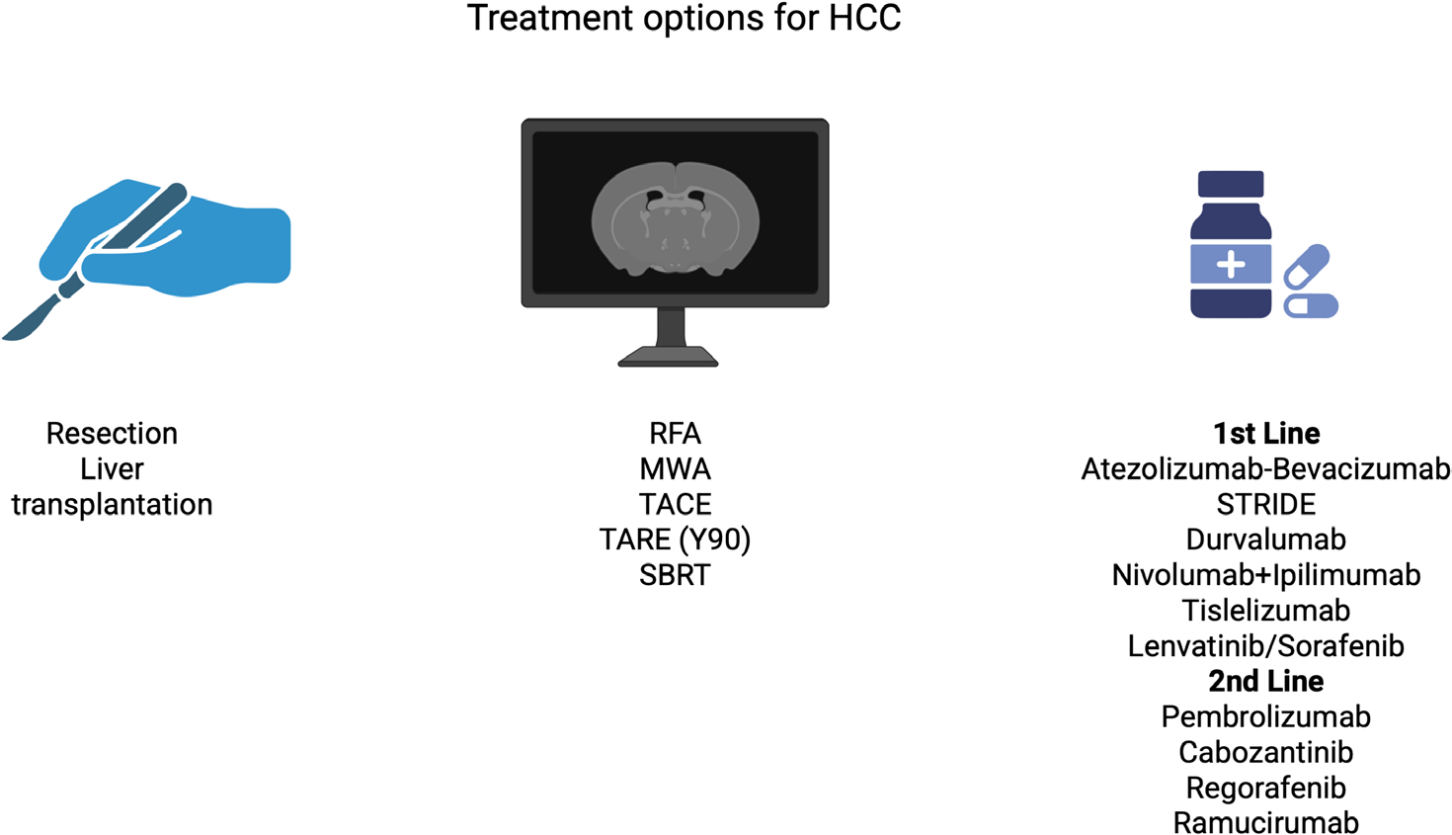
Available therapies for hepatocellular carcinoma. RFA, radiofrequency ablation; MWA, microwave ablation; TACE, transarterial chemoembolisation; TARE, transarterial radioembolisation; SBRT, stereotactic body radiation therapy.

Liver cancer is one of the leading causes of cancer‐related death worldwide, after lung and colorectal cancer [1]. Hepatocellular carcinoma (HCC) accounts for more than 85% of primary liver cancer, and despite advances in management, the age‐standardised death rate due to liver cancer is 5.95/100 000 population [2]. While viral hepatitis remains the most common cause of HCC globally, metabolic dysfunction‐associated steatotic liver disease (MASLD) is rapidly emerging as a leading aetiology of HCC, particularly in regions where viral hepatitis is declining due to effective prevention and treatment programmes [3]. Available treatment options for HCC include surgical interventions, locoregional interventions, immune checkpoint inhibitors (ICIs) and oral systemic therapies. (Figure 1) Traditionally, surgical resection, local ablation and liver transplantation (LT) are curative therapies for HCC. However, less than 20% of patients are deemed suitable for such curative therapies due to suboptimal surveillance, frequent late presentation, poor hepatic reserve and comorbid conditions [4, 5]. An effective national surveillance programme can aid in the early identification of HCC and likely lead to an increased proportion of patients receiving curative treatments for HCC.

With recent advances in the therapeutic armamentarium, including increasing data for multimodal approaches, the aim in unresectable HCC (uHCC) has shifted to optimising long‐term cancer‐free and overall survival, even among patients who are not amenable to curative treatments [6, 7, 8]. Further, recent evidence also suggests that multimodal management may bridge or downstage patients with advanced HCC to curative therapies. ICIs that have been proven efficacious for first‐line treatment for uHCC include atezolizumab‐bevacizumab (atezo‐bev), a single dose of tremelimumab plus repeated doses of durvalumab and nivolumab plus ipilimumab [9]. The Barcelona Clinic Liver Cancer (BCLC) staging system, which previously reported 10 months survival with systemic therapy, has now increased this estimate to more than 2 years [10, 11] due to the availability of these newer drugs [12, 13]. Accordingly, the use of ICIs has exponentially increased in recent years, being used in more than 70% of patients, and tyrosine kinase inhibitors (TKIs) now account for less than 10% of treated patients [14]. The advantages and disadvantages of each of these drugs have been enumerated in Table 1. In this review, we discuss the role of ICIs in the peri‐transplant period to enhance chances for a successful LT for advanced HCC while also decreasing the risk of recurrence post‐LT.

**TABLE 1:** Comparing the available systemic therapy in HCC in the context of LT.

	TKIs	ICI
1st line	Lenvatinib Sorafenib	Atezo‐bev STRIDE Single doses of Durvalumab Tislelizumab Nivolumab + Ipilimumab
2nd line	Cabozantinib Regorafenib	Pembrolizumab
Mechanism of action	Tyrosine kinase inhibition: anti‐angiogenic, inhibit tumour proliferation and tumour growth	Enhance immune system and lead to tumour death
Onset of action	Rapid	Slower, needs immune activation
Role of patients' immune system	Probably involved	Definitely involved
Tumour response after discontinuation of therapy (durability)	Rapid disease progression (less durability)	Delayed disease progression (more durability)
Ease of administration	Easy, outpatient therapy	Less convenient
Complete response	Rare (1%)	Not uncommon (~10%)
Data on combination with LRT	Strong	Evolving
Bridge to transplant	Rare	Feasible
Availability	Easy	Not universally available
5‐year survival	8%–10%	19%–25%
Post‐LT use	Yes, safe and are the first‐line drugs	Not recommended as they are associated with risk of rejection
Adverse events	Generally straightforward; manageable by most specialists	Often complex; requires multidisciplinary expertise and are time consuming
AEs of interest	Diarrhoea/proteinuria/fatigue/PPE/ nausea/vomiting/hypertension	Immune‐related AEs, bleed, Proteinuria, hypertension,

Abbreviations: AE, adverse events; ICI, immune checkpoint inhibitors; LRT, locoregional therapy; PPE, palmar‐plantar erythrodysesthesia; STRIDE, single dose of tremelimumab plus repeated doses of durvalumab; TKIs, tyrosine kinase inhibitors.

## 2 Methods

For this narrative review, we searched PubMed for relevant articles on ICIs and LT. The MeSH terms used were ‘liver transplantation’, ‘liver cancer, adult’ and ‘immune checkpoint inhibitors’. This strategy was supplemented by a recursive search of references from identified articles.

## 3 Evolution of LT for HCC

Due to poor selection strategies, initial studies reported a post‐LT survival of only 30% at 5 years for patients with HCC [15]. The landmark study by Mazzaferro and colleagues established the Milan criteria after reporting a recurrence‐free survival (RFS) rate of 83% at 4 years for patients with unifocal lesions ≤ 5 cm or 2–3 lesions, each ≤ 3 cm, without vascular or extrahepatic involvement [16]. However, only 20% of patients met the criteria, and among them, 80% underwent LT. Further, 56% of HCC patients planned for LT had received locoregional therapy (LRT) prior to LT. The study reported that up to one‐third of patients with HCC can be misclassified as within or outside Milan criteria [16, 17]. While practices vary across centres, Milan criteria still remain the benchmark comparator for newer criteria. To address the limitations associated with restrictive tumour size criteria and to broaden transplant eligibility, selection criteria for LT have progressively expanded over time while including biomarkers to address the biological behaviour [18, 19, 20, 21, 22, 23, 24, 25, 26, 27, 28] (Table 2).

**TABLE 2:** Criteria for LT for HCC.

Name	Criteria	Survival	Comments
Extended Toronto criteria [28]	Liver‐limited tumour irrespective of size and number with no cancer‐related symptoms (10 kg weight loss/increase in ECOG score ≥ 1 within 3 m), no vascular or biliary invasion and AFP < 500 ng/mL at LT	Actuarial survival of 78% at 5 years	68% had received LRT. Well suited for LDLT settings. Biopsy done to rule out poorly differentiated HCC
UCSF criteria [17, 19]	Single tumour ≤ 6.5 cm or ≤ 3 lesions with largest being ≤ 4.5 cm and total tumour diameter ≤ 8 cm	Survival of 75% at 5 years	18% exceeded the pathologic criteria of UCSF post LT and 5 year RFS was 59% in them. Multiple small tumours (sometimes incidental) are excluded
Milan criteria [16]	Single tumour < 5 cm or ≤ 3 tumours not more than 3 cm	75% actuarial survival and 83% RFS at 4 years	Considered as gold standard for best outcomes Too restrictive 27% misclassified as Milan had survival of 59% at 4 years. Pre‐LT embolisation survival was 79% vs. 69% in those who did not receive embolisation
5–5 criteria (Tokyo criteria) [24]	≤ 5 nodules none > 5 cm	3‐year RFS 94%	Criteria from LDLT settings. Derived from single centre
Upto 7 criteria [20] (Metroticket)	Size of largest tumour (cm) and with any additional lesions of 7 cm or less	5 year overall survival rate of 71.2%	28% misclassified as outside Milan criteria. Difficult to identify small tumours in cirrhotic liver. Derived from retrospective analysis based on Pathology
Asan criteria [22]	≤ 6 lesions none more than 5 cm and no gross vascular invasion	5 year recurrence rate 9% and survival at 5 years 76% Perioperative mortality 7%	Pre‐LT classification based on Milan and UCSF imaging criteria and post‐LT based on explant pathology. Valid for LDLT settings
Criteria's including tumour markers			
Kyoto criteria [21]	≤ 10 lesions with none > 5 cm and DCP (PIVKA) ≤ 400 mAU/mL	5 year survival rate 87% 5 year Recurrence rate 5%	74% had received LRT prior. Derived from LDLT settings DCP lab values variable and influenced by vitamin k antagonist/vitamin k injections. Viral dominant cohort
TTV/AFP criteria [18]	TTV ≤ 115 cm^3^ & AFP ≤ 400 ng/mL without macrovascular invasion or EHS	Survival 75% at 4 years	Dropout rate 42% for TTV/AFP vs. 25% for within Milan criteria. Lack of standardised tumour volume assessment across centres. Suited for centres with waiting time > 8 months. Study included those who were beyond criteria and were downstaged with criteria and were stable for 3 months
AFP‐French model [25]	Tumour size, number and log 10 of AFP	A score > 2 recurrence rate 50% and survival rate 47% at 5 years	
5–5‐500^23^	≤ 5 nodules none > 5 cm with a AFP ≤ 500 ng/mL	5‐year recurrence rate 7.3% Overall survival rate 76%	Developed in LDLT settings Need validation
Metroticket 2.0^26^	Tumour size, number and log 10 of AFP	AFP < 200 ng/mL and the sum of number and size of tumours < 7 AFP: 200–400 ng/mL+ size and number ≤ 5 AFP: 400–1000 ng/mL and size + number ≤ 4	Derived from large cohort. Valid for HCV‐related HCC Predicts 70% survival post LT
Seoul National University Hospital criteria (SNUH) [27]	Based on AFP levels and PET positivity	5‐year DFSR in low risk (AFP <200 ng/mL and PET[−]) was 86.1%; intermediate risk (AFP > 200 ng/mL or PET [+]) 79% and in high risk (AFP > 200 ng/mL and PET [+]) was 18.5%	Valid in recent years where utility of PET has increased especially in LDLT settings

Abbreviations: AFP, alpha‐fetoprotein; DCP, des‐ gamma carboxyprothrombin; ECOG, Eastern Cooperative Oncology Group; EHS, extrahepatic spread; HCC, hepatocellular carcinoma; hepatitis C virus; DFSR, disease free survival rate; LDLT, living donor liver transplantation; LRT, locoregional therapy; LT, liver transplantation; PET, positron emission tomography PIVKA, protein‐induced vitamin k absence; RFS, recurrence‐free survival; TTV, Total tumour volume; UCSF, university of California San Francisco.

## 4 Expanded Criteria and Downstaging Modalities

The main aim of LT in HCC is to provide long‐term RFS and overall survival. This goal can be achieved by establishing predictors of recurrence and offering LT for only those with favourable factors, as discussed above. There has been increasing recognition of the response to bridging or downstaging therapies while awaiting LT, which offer insights into tumour biology and are better predictors of post‐LT outcomes than the number and size of HCC at initial presentation. Bridging therapies are defined as those that keep tumours within Milan Criteria and reduce the risk of waitlist dropout (removal from transplant waitlist due to progression or deterioration in clinical status), whereas downstaging is defined as the use of therapies to decrease tumour burden from beyond to within criteria.

The most validated and universally accepted downstaging criteria in the West are the United Nation Organ Sharing Downstaging Criteria (UNOS‐DS). Patients exceeding Milan criteria but meeting one of the following are included for downstaging as per UNOS: single lesion 5.1–8 cm; 2–3 lesions none more than 5 cm or 4–5 lesions none more than 3 cm and with sum of maximal tumour diameter not more than 8 cm and none having extrahepatic or vascular spread. The UNOS‐DS criteria provide an excellent benchmark and are accepted worldwide for determining downstaging and LT [29].

## 5 LRTs As a Bridge to Transplant

LRTs have remained a mainstay of therapy for downstaging HCCs to within the preferred criteria and induce tumour necrosis, buy time and prevent tumour spillage intraoperatively (due to induction of necrosis and reduction in viable tumour cells) and reduce the risk of recurrence [30]. The type of LRT performed is dependent on the tumour number, size, location, hepatic reserve of the patient and local expertise. Local ablation is often used for single tumours less than 3 cm, whereas transarterial chemoembolisation (TACE) or transarterial radioembolisation (TARE) are often used for lesions > 4 cm [17]. A non‐randomised study reported that stereotactic body radiation therapy (SBRT), which involves more than 35 Gy doses, can also provide excellent pathologic response (in 60% of patients) and be used as a bridging modality [31]. LRT is most often useful in deceased donor settings, while such bridging therapies may be less relevant in living donor liver transplant (LDLT) settings, given the expedited time to transplant.

## 6 Advantages and Disadvantages of LRTs

The use of LRTs for bridging therapy must weigh the risk of dropout versus treatment‐related complications. Dropout occurs in more than 30% of patients who meet UNOS‐DS criteria, with a higher risk in those with larger tumour burdens or those with severe liver dysfunction (which can limit bridging therapy). However, LRTs can be associated with liver injury and treatment‐related hepatic decompensation (ascites, encephalopathy) in 10%–30% of patients [31, 32, 33]. More than 30% of patients drop out of the waitlist despite meeting UNOS‐DS criteria, and 3%–15% develop complications from downstaging and die from liver‐related events or decompensation [34]. Moreover, all LRTs demand specialised expertise for accurately identifying tumour location, precisely targeting correct lesions and determining optimal dosage, and are often associated with financial burdens. Lastly, tumour necrosis caused by LRTs, particularly radiation‐based therapies, may affect accurate assessment of tumour burden on pre‐transplant imaging [35]. Furthermore, it is unclear whether actuarial post‐LT RFS is affected by pre‐transplant therapy in patients satisfying Milan criteria [16]. Therefore, LRTs are preferred for patients outside of Milan criteria and/or when the transplant is not feasible in the near future, both in LDLT and DDLT settings. While many centres use LRTs in a majority of patients listed for LT, their benefit is best proven for downstaging patients outside of Milan criteria and bridging patients for whom the expected waitlist time exceeds six months [36].

## 7 ICI As a Bridging/Downstaging Modality to LT

Although LRTs have evolved and have become a standard of care for patients with HCC, ICIs may overcome some of the challenges and limitations of LRTs. In recent years, ICIs are emerging as effective modalities for downstaging or bridging patients with HCC to LT [37, 38, 39, 40, 41, 42, 43, 44, 45, 46, 47, 48, 49, 50] (Table 3). Use of ICIs can overcome the immune evasion mechanism of HCC to induce tumour regression and control the tumour burden in some situations where traditional therapies such as TKIs/LRTs were not beneficial (Table 4). HCC recurrence post‐LT is related to factors including microvascular invasion, larger tumour size, poor differentiation and unknown micrometastasis, which ICIs can target [51]. ICI induces potent T‐cell‐mediated anti‐tumour activity and activates memory T‐cells, which can target micrometastatic cells and microvascular invasions and theoretically reduce the risk of recurrence [41]. Currently, there are no set standardised criteria for patients who can be included for ICI therapy and planned for LT, and prospective studies are required to identify ideal candidates.

**TABLE 3:** Reported case series and data in literature on LT after ICI therapy.

Sl. no	Author, type of study, Country, Year	Inclusion criteria	Common ICI drugs used pre‐LT	Proportion of patients receiving LRT (and its details) prior to LT	All comers vs. DS criteria	Type of LT (LDLT vs. DDLT)	Time to LT	Explant features	Post‐LT outcomes reported in study (mortality, recurrence, ACR, others)	Predictors of rejection
1	Rezaee‐Zavareh et al. Multicentre retrospective. 2025^50^	Individual patient data meta‐analysis of studies reporting use of ICI prior to LT (91 cases from 30 studies)	Nivolumomab‐49.5% Pembrolizumab‐23.1% Atezolizumab+bevacizumab‐15.4% Others‐12.1%	77.8% (type not mentioned)	All comers (Within MC‐19%)		42 (71) days	Complete pathologic response‐33.8%	Mortality‐9.9% Rejection‐26.4% (only 1 AMR) Recurrence‐9.9%	Age and ICI washout period
2	Moeckli et al. Multicentre retrospective. 2025^48^	119 LT patients who received ICI prior to LT	Nivolumab‐32% Pembrolizumab‐23%; Atezolizumab‐20%	—	DS‐52% Bridging‐43%	DDLT‐77% (of 79) LDLT‐13% (of 79)	56 (30,122)	—	7.5% mortality and 21.8% recurrence; 20.1% developed ACR at 9 days post LT.	Interval between ICI and LT (< 50 days high risk) Pembrolizumab‐higher risk of ACR
3	Guo et al. Retrospective multicentre study. China. 2024 ^37^	83 patients with ICI who underwent LT	ICI + TKI + TACE in varying combinations	Yes (87%) had received TACE	82% as DS and 18% as bridging therapy	DDLT	58 (29–110) days		9.6% mortality at 8.1 months and 24% recurred at 5.5 months. Rejection increased mortality	27.7% after 11 days of LT 71.4% rejection if Time to LT < 30 days compared to 13% in < 30 days
4	Pang et al. Retrospective. China. 2024 [49].	121 patients who underwent LT for HCC	Various PD1 and PDL1 inhibitors	Yes (details not mentioned)	Patients within UCSF	DDLT	—	—	Mortality at day 90: 14.3% vs. 2.3% in non‐ICI Rejection: 23% vs. 5.8% in non‐ICI Infections post‐LT was 37% in ICI vs. 21% in non‐ICI group	ICI exposure Aetiology Shorter washout period of < 21 days
5	Kulkarni et al. Retrospective. India, 2024 [38].	12 of 115 (all comers) patients who achieved ORR after atezo‐bev therapy	Atezo‐bev	Yes in 34% of patients (Y90‐16.7%; Ablation‐8.3% and SBRT‐8.3%; None‐66.7%)	All comers of HCC who received atezo‐bev	LDLT and DDLT	89 (38–114) days	Complete pathologic response in 80%	20% post LT mortality. Rejection: 0% at 10 months. 33% waitlist mortality due to infections Wound healing complications	—
6	Lv et al. Pilot RCT. China. 2024^39^	22 patients with HCC outside Milan criteria	11 received pembrolizumab + Lenvatinib (PLENTY) and 11 received LRT	Control arm received TACE/RFA based in size	Outside Milan (DS)	DDLT	60.5 (25–193)	60% had tumour necrosis in PLENTY group	OS similar in by oth groups RFS was 70% in PLENTY compared to 30% in control group	
7	Tabrizian et al. Prospective multicentre study. USA. 2024 ^40^	117 HCC patients who received ICI with a intention to transplant	68‐Nivolumab 24‐atezo‐bev 21‐pembrolizumab 4‐STRIDE	94% received LRT (Y90‐41%; TACE‐36.8%; Ablation‐9.4%; Other‐6.8%)	31 remained within MC and 86 beyond	DDLT‐72.1% Salvage DDLT‐20.9% 7%‐LDLT	43 days (13–120)	24%‐complete necrosis 36%–50%–90% necrosis 40%– < 50% necrosis	Post‐LT survival 85% at 3 years. 50.4% drop out due to progression or death or non‐adherence 36.8% underwent LT	Time to LT from ICI < 3 monhts‐86% rejection compared to 14% in > 3 months
8	Lu et al. Retrospective. China. 2024 ^41^	159 patients who underwent LT (UCSF criteria) 39 ICI vs. 120 non‐ICI	Tislelizumab, Sintilimab, camrelizumab and atezolizumab	TACE‐43.4%; HAIC‐20.1%, RFA‐10.1%	Downstaging those outside UCSF	?DDLTs	50 (3–840) days Cycles‐ 4 (1–240)	—	No difference in RFS/OS. ICI improves OS in MVI patients. 23% AR in ICI compared to 5% in non‐ICI. Higher rejection related death in ICI group (12.8% vs. 0 in non‐ICI) Higher hospital stay in ICI group	Pre‐LT ICI increased risk of AR
9	Wang et al. Retrospective. China. 2023 ^42^	16 patients within UCSF criteria underwent LT after ICI	7‐Pembro 4‐sintilimab 2‐nivo and camrelizumab 1‐various	LRT‐100% (details not mentioned)	4 outside UCSF (downstage) and 12 within (bridging)	—	21 days for those who developed rejection and 60 days for those who did not	37.5% complete pathologic response	56.2% rejection (25% had BPAR) 31% recurrence rate	Most patients with pembro developed rejection. Plasma transfusions were protective
10	Chiang et al. Single centre phase 2 trial. China. 2023^43^	33 patients with advanced HCC (BCLC‐A/B‐36% and C‐64%)	Avelumab	TACE+SBRT‐100%	Downstaging intermediate tumours		—	—	55% amenable for curative therapy 12% underwent curative therapy (resection/ablation)	15% developed elevation in liver enzyme after TACE
11	Dave et al. Retrospective. USA. 2022^44^	8/86 waitlisted patients had received ICI	—	Yes (details not mentioned)	Bridging in 7 patients and downstaging in 1	DDLT in 5 patients in ICI vs. 47/78 in non‐ICI group	105 days (11–354)	Lower viable tumours in ICI group than non‐ICI group	40% rejection 40% graft loss Waitlist mortality‐0%	Use of ICI
12	Qiao et al. Retrospective. China. 2021^45^	7 LT recipients who had received ICI prior	Pembrolizumab or Camrelizumab + lenvatinib	—	—		42 days	—	14.3% rejection	IL‐17 decreased, CD4/CD8 and CD3/CD8 ratio increased in patient who developed rejection
13	Chen et al. Retrospective. China. 2021^46^	5 LT recipients who were outside of MC and UCSF	Nivolumab	TACE‐100%, RFA‐20%	Downstaging	DDLT	63.8 ± 18.26 days	20% complete response. Rest had massive necrosis	None developed rejection. 40% recurrence within 1 year	—
14	Tabrizian et al. Retrospective. USA. 2021^47^	9 patients who underwent LT after ICI	Nivolumab	LRTs‐100% (no details provided)	Bridging (all within UCSF)	8‐DDLT 1‐LDLT (55% salvage LT)	90% with 1 month of last dose	Complete tumour necrosis in 30% of patients	11.1% mild rejection No mortality	Due to Tac trough levels < 6 ng/mL

Abbreviations: ACR, acute cellular rejection; BPAR, biopsy proven acute rejection; DDLT, deceased donor liver transplantation; DS, downstaging; HCC, hepatocellular carcinoma; ICI, immune checkpoint inhibitor; ICI, immune checkpoint inhibitors; LDLT, living donor liver transplantation; LRT, locoregional therapy; LT, liver transplantation; MC, Milan criteria; ORR, objective response rate; PDL, programmed death ligand; RCT, randomised controlled trial; RFA, radiofrequency ablation. SBRT, stereotactic body radiation therapy; TACE, transarterial chemoembolisation; TKI, tyrosine kinase inhibitor; UCSF, university of California San Francisco.

**TABLE 4:** Comparison of available modalities for downstaging.

	LRT alone	ICI alone
Recommended stage of BCLC for use	BCLC 0,A,B	BCLC C
Evidence	Robust	Upcoming
Mechanisms	Direct tumour killing by cytotoxic effect or blocking blood supply	Inhibits immune checkpoints and enhances systemic immune response to cancer cells
Effect	Local on tumour tissue	Systemic effect can kill tumour tissue including distant metastasis
Effect on CTCs	None	Moderate
Recurrence rates	High pre‐LT (both local and extrahepatic) No effect on post LT	Low pre‐LT Probably increased post‐LT
Combination with TKIs	Enhanced tumour control	No effect
Combination of LRT and ICI	Increased expression of neoantigens and may increase chance of tumour control	
Assessment of response post therapy	Radiological	Radiological
Adverse events leading to delisting	Risk of decompensation in 30%	Infections in 30% of patients Autoimmune diseases in upto 10%
Washout period	Not required	Required (1–3 months)
Risk of rejections	Similar to patients not receiving LRT	May be increased
Expertise for administration	Required	Required

Abbreviations: BCLC, Barcelona Clinic Liver Cancer; CTC, circulating tumour cells; ICI, immune checkpoint inhibitor; LRT, locoregional therapy; LT, liver transplantation; TKIs, tyrosine kinase inhibitors.

## 8 Challenges With ICI Pre‐LT

Although generally well tolerated, immune‐related adverse events leading to liver failure (in 1%–2% of patients) and increased risk of infection due to T‐cell‐mediated autoimmunity, impaired Treg and TH17 function, and risk of delisting remain a significant concern with ICI therapy [52, 53]. Infections post‐ICI are common in patients with diabetes mellitus, those receiving corticosteroids and those receiving a combination of ipilimumab and nivolumab [54, 55]. Infections in patients with HCC who receive ICI range from uncomplicated bacterial infections to rare opportunistic infections, viral infections and tuberculosis reactivation [54, 56, 57]. Infections are reported in 10%–50% of patients receiving ICI, with up to 33% developing infection‐related liver failure [38, 56, 58]. While infections have not been reported to impact overall survival, they may affect quality of life and contribute to morbidity [56]. Infections usually occur in the first six months of initiation of treatment but are not uncommon even after stopping the ICI and are associated with increased risk of infection‐precipitated liver failure [38, 54]. Such patients may undergo salvage LT (after adequate infection control), which has been reported to provide a survival benefit. A second challenge is the risk of decompensation, which is reported in up to 25% of patients who received ICI [59]. Lastly, adverse ICI‐related major adverse cardiovascular events are reported in 7% of patients and treatment‐related discontinuation is reported in up to 15% [60, 61].

A multicentre study including 83 patients who underwent LT after ICI therapy reported 24% HCC recurrence within a median duration of 5.5 months (4.2–14.1) post‐LT [37]. The high recurrence rate was likely due to the inclusion of patients with both stable and progressive disease [37]. It is recommended to include only those with complete or partial responses to ICI for LT^42^, although some studies suggest including patients with stable disease [39]. However, radiological evaluation is prone to misclassification of response, especially in patients receiving ICIs, where radiological pseudoprogression and hyperprogression (HPD) post‐ICI are common and can impede appropriate assessment of response in 15%–20% of patients [62]. A individual patient data meta‐analysis including 91 patients who underwent LT after ICI reported that patients who receive four or fewer cycles of ICI pre‐LT (due to inadequate tumour control or insufficient washout period), those beyond Milan criteria and those with micro‐ or macrovascular invasion was at increased risk of HCC recurrence post‐LT, and such patients should not be offered LT [50].

Rejection is the most feared complication following LT in patients who have received prior ICIs. Most rejection episodes occur within the first month of LT, and the earliest reported rejection episode leading to fatal hepatic necrosis due to nivolumab was on day 3, when the recipient did not achieve normalisation of hepatic biochemical tests post‐LT [37, 63]. The incidence of acute cellular rejection episodes in patients receiving ICI (pre‐LT) is 20%–23% compared to 6% in those who have not had prior ICI exposure [44, 49]. These events are usually severe, less responsive to immunosuppression (< 50% reversal rate) and are associated with higher healthcare burden, graft loss and mortality [41]. The only treatment in such severe cases can be a rescue re‐transplantation, as none of the therapies (including anti‐thymocyte globulin, IVIG, rituximab and plasmapheresis) successfully treats these severe rejection episodes [40, 63, 64].

**FIGURE 2:**
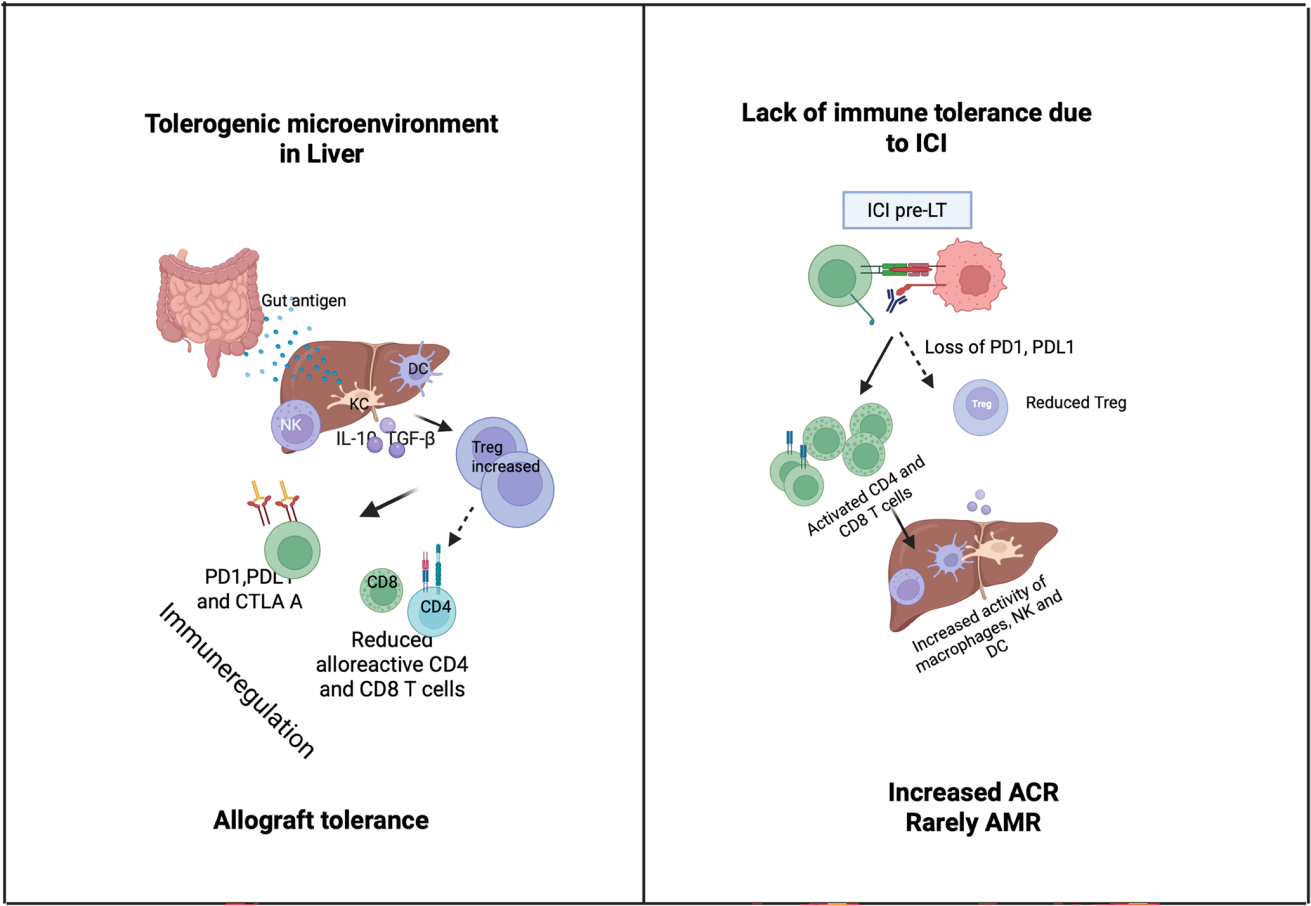
Mechanisms of immune tolerance and ICI‐associated rejection in LT. The liver is an immunologically privileged organ that promotes immune tolerance through constant exposure to gut‐derived antigens and the presence of tolerogenic cells such as Kupffer cells, dendritic cells and liver‐resident natural killer (NK) cells. These cells produce anti‐inflammatory cytokines (IL‐10, TGF‐β) and induce regulatory T‐cells (Tregs), maintaining graft tolerance. In contrast, immune checkpoint inhibitors (ICIs) disrupt this balance by enhancing CD8^+^ effector T‐cell responses and reducing Tregs, leading to increased activity of CD4^+^ T‐cells, macrophages and NK cells. This immune activation lowers graft tolerance and contributes to acute cellular rejection in liver transplant recipients. PD‐1, PD‐L1 and CTLA‐4 pathways, which are key to maintaining allograft tolerance, are blocked by ICIs, further exacerbating immune‐mediated injury. Created in BioRender. Kulkarni, A. (2025) https://BioRender.com/0lymdyb.

While few reports of antibody‐mediated rejection episodes have been reported, acute cellular rejection episodes are more common than the former due to the significant role of T‐cells in ICI recipients [64]. The mechanism of immune tolerance and graft rejection due to ICI is depicted in Figure 2.

## 9 Challenges With ICI Post‐LT

Few studies have reported the use of ICI in the post‐LT period [65, 66]. Table 5 shows the literature on the use of ICI in the post‐LT period [66, 67, 68, 69]. The most common indication for the use of ICI is melanoma, in which the response rate is 50% compared to none in patients who received ICI for recurrent HCC [68]. However, the rejection rate in such patients is more than 60%, with a mortality rate of 50% in patients who received ICI post‐LT [45, 65]. A meta‐analysis suggested that using ICIs prior to 3 years after LT may increase the risk of rejection episodes, which is related to the presence of PD‐L1 on the graft [70]. However, a small case series has reported graft rejection even if ICI is used four years after LT [69]. ICIs are associated with unique adverse events such as immune‐related adverse events (iRAE), colitis and proteinuria, which may interfere with immunosuppression drug management [52, 67]. Current evidence does not support the use of ICIs in the post‐LT period.

**TABLE 5:** Post LT use of ICI and outcomes.

Author, Type of study, Country, Year	N	Indication for ICI	Time from LT to ICI	ICI used	Outcomes (response, mortality, duration of therapy, rejections)
Rudolph et al. Retrospective chart review, USA, 2023^69^	4 post LT patients received ICI	HCC‐2 Small bowel adenocarcinoma‐1 Rectal SCC‐1	114 months	Atezo‐bev‐2 Nivolumab‐2	None responded Death after 5.6 months of ICI
Owoyemi et al. Retrospective database analysis. USA, 2022^67^	8 post LT patients received ICI	HCC‐5 Melanoma‐2 SCC‐1	1.6 years (0.7–3.5)	Nivolumab‐6 Pembrolizumab‐2	Rejection‐25% Disease progression‐63% Death‐75% Duration of therapy was 2.8 months
Abdel‐Wahab et al. Systematic review including retrospective data analysis. USA, 2019^66^	11 post LT patients received ICI	Melanoma‐6 HCC‐4 NSCLC‐1	< 5 years	Nivolumab‐4 Pembrolizumab‐4 Ipilimumab‐3	Response (CR/PR)‐27.2% Progression‐54% Rejection‐36% Graft loss‐27.8% Mortality‐27.8% iRAE‐21% Mortality high in graft rejection patients
DeLeon et al. Retrospective data analysis. USA, 2018^68^	7 post LT patients received ICI	HCC‐5 Melanoma‐2	3.1 years	Nivolumab‐5 Pembrolizumab‐2	28.5% rejection Zero response rate in HCC 50% complete response in melanoma patients

Abbreviations: CR, complete response; CR, complete response; HCC, hepatocellular carcinoma; ICI, immune checkpoint inhibitor; iRAE, immune‐related adverse events; LT, liver transplantation; mTORi, mammalian target of rapamycin inhibitor; NSCLC, Non‐small cell lung cancer; PR, partial response PR, partial response; SCC, squamous cell carcinoma.

## 10 Prevention of Rejection and Other Complications Post‐LT

Several studies have reported a significant correlation between the time interval between the last dose of ICI to LT and rejection episodes, suggesting lower intervals to be associated with an increased risk of rejection. The half‐life of the currently available drugs, such as durvalumab, pembrolizumab, nivolumab and atezolizumab, ranges between 18 and 28 days, with the shortest half‐life being 5 days for camrelizumab and 15 days for ipilimumab [71]. More than 70% develop rejection if the interval is less than 30 days, compared to 12% with longer intervals [37, 49]. A systematic review recently suggested that the risk of rejection can be reduced to ≤ 20% (a number comparable to those without ICI exposure) if the time from ICI to LT is at least 90 days or more [50]. Therefore, the suggested minimum time to LT after the last dose of ICI should be at least 52 days, if not greater than 90 days [48, 72]. Protocolised liver biopsies at frequent intervals may be helpful to identify graft rejection early on [73].

Intraoperative immunosuppression varies across centres, with some centres using basiliximab and corticosteroids, while few centres have reported using only corticosteroids [37, 38, 47]. However, post‐LT patients with HCC should ideally receive a triple‐drug combination of corticosteroids, calcineurin inhibitors and anti‐metabolites for the initial period, followed by the addition of antiproliferative agents (mTOR inhibitors) for maintenance after 1 month [38, 74]. The median tacrolimus levels in those who developed rejection were 7.1 μg/L which could be reversed by doubling the tacrolimus trough levels to 15 μg/L. [42] Therefore, we suggest maintaining tacrolimus levels of 12–15 μg/L in the immediate post‐LT period in patients who receive ICIs pre‐LT. Since T‐cells play a major role in graft rejection, it is unknown whether the use of T‐cell‐specific antibodies (daclizumab, basiliximab, muromonab‐CD3, alemtuzumab or anti‐thymocyte globulin) for induction may reduce the risk of rejection by eliminating T‐cells induced by ICIs pre‐LT [75]. In cases where patients undergo ABO‐incompatible transplants, measures such as the use of plasmapheresis, basiliximab and prophylactic mycophenolate mofetil to suppress T‐cell activity have been shown to reduce the risk of rejection [76]. Further, plasma transfusions in a small case series have been reported to be protective against rejection by diluting circulating PD1, and a case report also suggested no rejection episode in a patient who underwent ABOi living donor LT post‐ICI after plasmapheresis [42, 77]. Whether prophylactic plasmapheresis or excessive plasma transfusions in such patients with prior ICI exposure who undergo LT have reduced risk of rejection episodes and better outcomes needs to be assessed.

Wound complications due to the use of VEGF inhibitors are reported and need careful examination to prevent sepsis and wound dehiscence [38]. Clinical examination for wound‐related complications and regular follow‐up with hepatic biochemical tests is a must for the first month [46].

## 11 LRTs And ICI

ICIs alone have been reported to provide an objective response rate of 20%–25% [13, 52]. The addition of LRTs can further enhance the expression of antigens and induce the release of proinflammatory cytokines, providing ICIs with a broader scope of action and a more robust immune response [78]. The addition of VEGF inhibitors can augment favourable T‐cell infiltration and prevent neovascularisation. For patients within and beyond the Milan criteria, the combination of ICI and LRTs provides potential benefit. More than 90% can be downstaged with combination therapies compared to 60%–70% in the ICI alone group. Therefore, the combination of ICI and LRTs can be considered for patients with macrovascular invasion and large tumours to improve survival and downstage them to LT criteria. A single‐arm phase 2 trial has also reported more than 50% improved amenability of curative therapies for patients with locally advanced uHCC (BCLC C) who received a combination of TACE, SBRT and avelumab [43]. In the recent EMERALD‐1 study, the combination of durvalumab + bevacizumab and TACE increased the median progression‐free survival (15 months vs. 10 months for TAVE + durvalumab vs. 8.2 months for TACE alone) for patients with TACE‐eligible uHCC with Child‐Pugh score < B7 [79]. However, the combination was associated with an increased risk of grade 3/4 adverse events and a higher proportion of patients discontinued therapy due to adverse events in the combination arm [79]. Similarly, the LEAP 012 trial reported that the combination of lenvatinib, pembrolizumab and TACE resulted in a progression‐free survival of 14.6 months, compared to 10 months in the TACE plus placebo arm [80]. Survival data remains incomplete at this time, but there was a trend towards improved survival at the first interim analysis (hazard ratio, 0.8 [0.57–1.11]). However, 71% of patients in the combination groups developed grade 3 adverse events compared to 32% in the TACE‐only arm. The combination therapies of ICI and LRT have the advantages of enhanced anti‐tumour effect, better response rate and potential to convert an unresectable tumour to resectable or curative LT; nevertheless, such combination therapies increase the risk of treatment‐related adverse events, decompensation and liver failure, meriting frequent monitoring and higher healthcare burden.

## 12 LDLT Vs. DDLT for HCC

Approximately 30% of LDLTs in Asia are being performed for HCC [30]. In contrast, the number of transplants (majority DDLT) performed for HCC in the USA has decreased from 20% in 2012 to 11% in 2022 [81]. The probable reason for this is the modification in MELD exception criteria, which led to a 12% decrease in transplants for HCC in 2022 compared to 2012 but with an excellent graft survival of 77% at 5 years [81]. The concerns with respect to pre‐LT ICI include the timing of LT post‐ICI therapy, with a washout period of at least 3 months being associated with a reduced risk of post‐LT rejection. In the West, the allocation is based on the MELD exception points for patients with HCC who remain stable for 6 months to avoid the inclusion of patients with aggressive tumours who are predisposed to increase the risk of recurrence post‐LT [82]. It is well known that a waiting time of more than 9–12 months increases the hazard of waitlist dropout and mortality by 60% in patients without live donors who wait for cadaveric liver, [83] while the presence of living donors reduces the risk of death by 33% in waitlisted patients and waitlist time by 1.5–2 months [83]. A timely LDLT in an ideal candidate can reduce the risk of waitlist mortality and achieve post‐LT outcomes similar to DDLT [84]. Therefore, LDLT has the advantage of lower waitlist mortality, and transplants can be timed based on patients' clinical status and timing from the last ICI therapy. While LDLT plays a critical role in regions with limited access to deceased donor organs, ethical concerns may arise in certain settings where institutional or individual incentives to increase transplant volumes could unintentionally influence candidate selection. This may increase the risk of offering LDLT to marginal candidates who might derive limited long‐term benefit, underscoring the importance of stringent selection criteria and ethical oversight. Furthermore, the inclusion criteria for LT in Asia vary from centre to centre and are mainly surgeon‐dependent (unlike Milan/UCSF in the West). Therefore, the International Liver Transplant Society (ILTS) recommends post‐transplant survival to be at least 60% at 5 years for patients undergoing LDLT and a Clavien‐Dindo grade I/II complication rate of less than 20% and III/IV of less than 5% for donors as a benchmark [85]. These outcomes are achievable in patients who do not have vascular invasion but not in patients with major vascular invasion [86, 87]. Instead of fast‐tracking patients for LDLT, it seems reasonable to downstage such patients prior to LT. Lastly, due to a lack of unified organ allocation programmes, organ scarcity and poor acceptance of LT in Asia, a good proportion of centres perform salvage LT for patients with prior resection who develop recurrence or for those who develop liver decompensation post‐LRTs. Salvage LT provides excellent oncological outcomes even in such patients [88].

## 13 Future Directions

### 13.1 Pre‐LT

Most patients who have undergone LT after ICI are mostly HBV‐related HCC patients. It is unknown whether this is related to HBV being the most common cause globally, more aggressive surveillance and LT patterns in Asia, or due to a better response with ICI in HBV‐related HCC, which has a better immune profile than MASLD‐related HCC [89]. Increasing data show that predictors of waitlist dropout include a doubling of alpha‐fetoprotein (AFP) levels from initial levels and being beyond MC, while complete or partial response leads to decreased chances of dropout [40]. Further studies are required to identify the role of novel tumour biomarkers such as AFP‐L3, DCP and circulating tumour cells in predicting waitlist dropout and tumour recurrence post‐LT. Liquid biopsy has been utilised for identifying candidates for LT [90]. Next‐generation sequencing for genetic mutations and tumour mutational burden prior to LT can identify ideal candidates for LT [91]. Patients with aggressive mutations such as TP53, TERT promoter mutation and CTNNB1 who are at risk of high tumour burden and extrahepatic metastasis should not be considered for LT [38].

Infections are common in patients with HCC who receive ICI, and the role of long‐term albumin infusions (immunomodulator) and other non‐antibiotic measures to prevent infections is needed [92]. The role of blood‐based biomarkers of tuberculosis (TB) to identify latent TB infections, especially in endemic regions, and drug interactions of pre‐emptive antitubercular therapy in patients with HCC planned for ICI needs assessment to prevent TB reactivation [93]. The role of plasmapheresis prior to LT and plasma transfusions during surgery, which may lead to dilution of PD‐1 antibodies and probably avoid episodes of rejection post‐LT, needs to be evaluated. The combination of ICI and LRTs enhances the likelihood of curative therapies such as LT, resection or ablation. However, only a small subset undergo LT, and the majority remain under observation [43]. The literature on the natural history and management of such patients who achieve cure with ICI with or without LRT is unclear.

### 13.2 Post‐LT

PD‐L1 expression on liver grafts of patients who developed graft rejection has been reported but needs further confirmatory studies in a large cohort [68, 94]. Although transplant immunology is intriguing, the role of protocolised liver biopsies and assessment of the CD4/CD8 ratios, tumour infiltrating lymphocytes and PD‐L1 expression post‐LT for prediction of graft rejection is yet to be evaluated. Furthermore, it is well known that PD‐1 occupancy on circulating T‐cells remains to be more than 70% even at 2 months post‐single dose of ICI, and the role of liquid biopsy in predicting rejection episodes needs to be assessed [95]. IL‐17, CD4/CD8 and CD3/CD8 ratio are known to predict rejection episodes in patients who undergo LT after ICI [45]. Several immunological biomarkers have been explored to assess rejection risk and immune activation in LT recipients receiving ICIs. These include PD‐L1 expression in graft tissue, peripheral T‐cell exhaustion markers (e.g., PD‐1, TIM‐3), donor‐derived cell‐free DNA (dd‐cfDNA) and proinflammatory cytokines such as CXCL10 and IFN‐γ [96, 97]. While not yet validated for routine clinical use, these biomarkers may aid in risk stratification and early detection of graft rejection. Such novel non‐invasive biomarkers to identify rejections are required. Recombinant VEGF infusions to promote wound healing need to be assessed in pre‐clinical and clinical settings. Although there are some encouraging reports on the use of ICI in the post‐LT period, further research is needed to understand safe timing and effective drugs in the post‐LT period. There is extensive research underway on combination strategies for HCC, which are shown in Table 6.

**TABLE 6:** Trials on ICI and LT.

Study details	Type of study	Interventions	Outcome	Status
NCT05717738	Multicentre ambispective study	TACE + ICI + TKI pre‐LT	Number of patients amenable for curative therapy including LT	Recruiting
NCT05713994	Observational	HAIC + ICI + TKI	Number of patients amenable for curative therapy including LT	Recruiting
NCT05475613	Phase 2 Interventional	LRT + PD‐1 Inhibitor (downstaging protocol for beyond Milan patients)	Post‐LT event free survival	Recruiting
NCT05411926	Observational (retrospective)	PD‐1/PD‐L1 in pre‐LT patients in those who underwent LT	Acute rejections	Unknown
NCT05027425	Phase 2 interventional	STRIDE in LT listed patients	Acute rejection rate	Recruiting
NCT06725121 (MACRO‐TRANS)	Phase 1/2	SBRT or Y90 + atezo‐bev for HCC with main PVT pre‐LT	Number of patients amenable for LT	Recruiting
NCT06254248 (IMMUNO‐TH)	Phase 2 interventional	Atezo‐bev for recurrent HCC 6 months post‐LT	Rejection rate	Not yet recruiting
NCT05913583	Retrospective observational	ICI vs. non‐ICI patients who underwent LT	Rejection at 1 year	Recruiting
NCT05879328 (ImmunoXXL)	Prospective observational	Atezo‐bev in patients with AFP adjusted up to 7 criteria	Proportion undergoing LT	Recruiting
NCT05063565 (ROWAN)	Prospective observational	TheraSphere Y90 + STRIDE	Objective response rate and proportion undergoing LT	Recruiting

Abbreviations: HAIC, hepatic arterial infusion chemotherapy; HCC, hepatocellular carcinoma; ICI, immune checkpoint inhibitor; LT, liver transplantation; PD, programmed death; PVT, portal vein thrombosis SBRT, stereotactic body radiation therapy; STRIDE, single dose of tremelimumab plus repeated doses of durvalumab; TKI, tyrosine kinase inhibitor.

## 14 Conclusion

**FIGURE 3:**
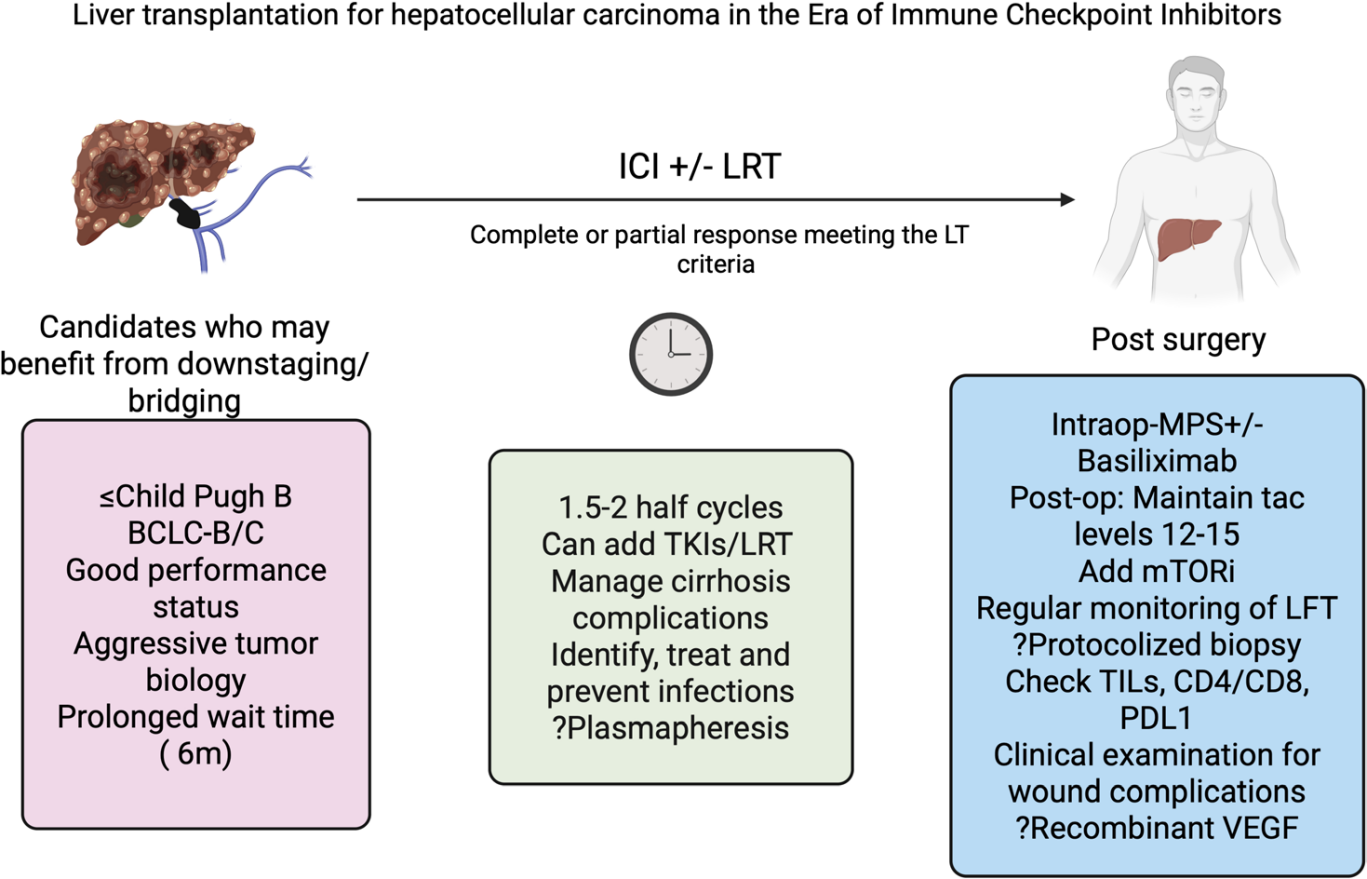
Liver transplantation for hepatocellular carcinoma (HCC) in the immune checkpoint inhibitor (ICI) era. CTP, Child Turcotte Pugh; TKI, tyrosine kinase inhibitors; LDT, locoregional therapy; MPS, methylprednisolone; mTORi, mammalian target of rapamycin inhibitor; LFT, liver function test; TIL, tumour infiltrating lymphocytes; PDL1, programmed death ligand; VEGF, vascular endothelial growth factor. Created in BioRender. Kulkarni, A. (2025) https://BioRender.com/b27a451.

The management of HCC is rapidly evolving with recent discoveries of newer systemic therapies, such as ICIs, which can downstage advanced HCC and provide excellent long‐term post‐LT survival. Despite several extended criteria being helpful in identifying ideal candidates for LT, the Milan criteria remain the gold standard. The ethical challenges of graft allocation for patients who have been downstaged with ICI have to be balanced with others with advanced liver failure, given the risk of graft rejection and post‐LT recurrence in such downstaged patients. Nevertheless, ICIs have brought a ray of hope for patients with advanced HCC who can be downstaged to LT (Figure 3).

## Author Contributions

**Anand V. Kulkarni:** conceptualization, writing – original draft, methodology, data curation. **Amit G. Singal:** supervision, writing – review and editing, validation. **K. Rajender Reddy:** conceptualization, writing – review and editing, supervision, validation.

## Conflicts of Interest

Amit Singal has served as a consultant or on advisory boards for Genentech, AstraZeneca, Eisai, Bayer, Exelixis, Merck, Elevar, Boston Scientific, Sirtex, FujiFilm Medical Sciences, Exact Sciences, Helio Genomics, Roche, Glycotest, Abbott, DELFI, IMCare, Mursla, Curve Bio and Universal Dx. K.Rajender Reddy declares research funding received from Mallinckrodt, Exact Sciences, BMS, Intercept, Merck, Gilead, Grifols, Sequana, HCC–TARGET, NASH–TARGET, BioVie, Regeneron, Camarus, GSK, B and D Instruments which were paid to the institution. K.R.R. has served on the Advisory Board of Spark Therapeutics, Mallinckrodt, Novo Nordisk, Novartis, Astra Zeneca, Arbutus, Genkyotex, Pfizer, Bio Vie and Genfit. K.R.R. also serves as an associate editor for Gastroenterology and receives compensation. K.R.R. is also a co‐chair of the Task Force for COVID activities of AASLD (No compensation).

## Data Availability

Data sharing not applicable to this article as no datasets were generated or analysed during the current study.
